# Supplementary material for: Tooth enamel nitrogen isotope composition records trophic position: a tool for reconstructing food webs
Source: Commun Biol. 2023 Apr 7;6:373. doi: 10.1038/s42003-023-04744-y (PMC10082005; doi:10.1038/s42003-023-04744-y)
Supplement: Supplementary file 2 — Supplementary Materials [file 42003_2023_4744_MOESM2_ESM.pdf]

Supplementary Materials for

Tooth enamel nitrogen isotope composition records trophic position: a tool for reconstructing food webs

Jennifer N. Leichliter[1,2,3]*, Tina Lüdecke[1,2,4]*, Alan D. Foreman[1], Nicolas Bourgon[5], Nicolas N. Duprey[1], Hubert Vonhof[6], Viengkeo Souksavatdy[7], Anne-Marie Bacon[8], Daniel M. Sigman[9], Thomas Tütken[3], Alfredo Martínez-García[1]

[1]Organic Isotope Geochemistry Group, Climate Geochemistry Department, Max Planck Institute for Chemistry, 55128 Mainz, Germany.

[2]Emmy Noether Group for Hominin Meat Consumption, Max Planck Institute for Chemistry, 55128 Mainz, Germany.

[3]Institute of Geosciences, Department of Applied and Analytical Paleontology, Johannes Gutenberg University, 55128 Mainz, Germany.

[4]Senckenberg Biodiversity and Climate Research Centre, 60325 Frankfurt, Germany.

[5]Department of Human Evolution, Max Planck Institute for Evolutionary Anthropology, 04103 Leipzig, Germany.

[6]Inorganic Gas Isotope Geochemistry Group, Climate Geochemistry Department, Max Planck Institute for Chemistry, 55128 Mainz, Germany.

[7]Department of Heritage, Ministry of Information, Culture and Tourism, 0100 Setthathirath Road, Vientiane Capital, Lao People's Democratic Republic.

[8]Université de Paris, BABEL CNRS UMR 8045, Paris, France.

[9]Department of Geosciences, Princeton University, Princeton, New Jersey 08544, U.S.A.

* Jennifer Leichliter and Tina Lüdecke, these authors contributed equally to this work
Email: Jennifer.Leichliter@mpic.de, Tina.Luedecke@mpic.de

This PDF file includes:

Supplementary Discussion
Supplementary Figures S1 to S7
Supplementary Tables S1 to S4
Supplementary References

Supplementary Discussion

Tooth Formation

To reconstruct adult diet, and avoid the isotopic effect of breast milk consumption[1-4], the teeth that mineralize latest in life were targeted in this study whenever possible (Tab. 1). In most mammals, the latest forming tooth is the third permanent molar. In this study only elephants, felids (lions and leopards), canids (African wild dogs), and hyenas differ in their tooth formation.

African elephants have a total of six successively developing molars (M1 – M6) in each dental quadrant which are shed throughout their lifetime as they wear. For this study we sampled the M4 or later forming molars, (i.e., 7 years of age and older[5]) which erupt after the individuals are fully weaned[6].

In addition, the M1 or M2 of one juvenile elephant was sampled, but was not included in any statistical analyses. These teeth develop between birth and 2 years of age[5], so this individual would have still relied heavily on breastmilk at the time that it died (weaning in African elephants occurs between 3.5 to 6 years of age[6]), thus its $\delta^{15}N_{enamel}$ value likely incorporates a weaning signal. Tooth enamel and mandibular bone would have formed at about the same time in this individual. This is confirmed by similar $\delta^{15}N$ values ($\delta^{15}N_{enamel}$ = 10.7‰; $\delta^{15}N_{collagen}$ = 10.2‰) in this juvenile individual.

Felids have only one molar (M1), which is the latest forming tooth and was therefore sampled for this study. Lions are usually weaned at ca. 6 to 7 months, and enamel mineralization of adult teeth begins at 9 to11 months, and is concluded by 28 to 36 months[7-9]. Little information is available regarding the rate of tooth enamel mineralization in modern leopards, but canines, molars, and premolars erupt between 8 to12 months, about 5 months after weaning[10]. Thus, the stable isotope data reported for felids should reflect adult diet.

African wild dogs are generally weaned at a younger age (35 days) than are most other carnivores, thus their permanent molars mineralize when he animal is consuming an adult diet[11].

In spotted hyenas, the changeover from deciduous to permanent dentition usually takes place between 12 and 14 months [12], with tooth mineralization taking place in the months before eruption (i.e., 11 to 13 months). Hyenas are usually weaned after 12 months, thus permanent teeth mineralize while cubs are still nursing[12]. In this study, hyena $\delta^{15}N_{enamel}$ values are significantly higher than those of other carnivores (p = 0.011), likely as a result of breastmilk consumption during enamel mineralization. In contrast, hyena $\delta^{15}N_{collagen}$ values do not differ from those of the other carnivores (p = 0.990) indicating that death occurred after weaning, when individuals were consuming an adult diet. If hyenas are excluded completely from the dataset the correlation between $\delta^{15}N_{enamel}$ and $\delta^{15}N_{bone\text{-}collagen}$ increases to 0.78.

In conclusion, adult diet is reflected for nearly all taxa in this study (with the exception of the juvenile elephant and hyenas).

Nitrogen isotope variability in tooth enamel

The $\delta^{15}N$ values of herbivore body tissues are believed to be determined primarily by the isotopic composition of their diet[13-18], which is in turn controlled by abiotic factors specific to each ecosystem. It has been well demonstrated, for instance, that a negative relationship between plant $\delta^{15}N$ values and rainfall exists at both global and regional scales[19-21]. Plants growing in arid regions tend to have elevated $\delta^{15}N$ values relative to plants growing in regions that experience

greater rainfall. The $\delta^{15}N$ values of plants are in turn passed along to the herbivores that consume them.

Herbivores:

In general, the $\delta^{15}N$ values of herbivore body tissues also correlate negatively with rainfall[17,22-26]. However, data from some studies of African mammals recorded unexpectedly high $\delta^{15}N$ values for herbivores living in hot and arid environments, often exceeding the expected degree of trophic ^{15}N enrichment based on rainfall amounts and plant $\delta^{15}N$ values. These studies noted that when the same taxa occurred in different habitats, $\delta^{15}N$ values were greater (by 1 to 3‰) in the tissues of individuals living in open and dry habitats (i.e., savannas) than in more closed, wet habitats (i.e., forests)[17,22-25,27]. This enrichment effect has been hypothesized to be the result of physiological adaptations for water conservation. Proposed mechanisms include increased protein catabolism in drought-tolerant taxa[17], increased reliance on amino acids produced by symbiotic gastrointestinal bacteria in arid regions where plant nitrogen content is low[23], and greater utilization of amino acids from hydrolyzed urea[28]. However, when herbivorous taxa in this study were grouped according to water dependence (high, low, and none; after Hempson et al.[29]; see Table 1), we found no statistically significant differences in $\delta^{15}N_{enamel}$ ($\chi^2(2) = 3$, $p = 0.223$; see Fig. S6) between groups, suggesting that water dependence does not drive the observed variation in $\delta^{15}N_{enamel}$.

Carnivores:

In the carnivore $\delta^{15}N_{enamel}$ dataset, the spotted hyenas exhibited particularly elevated $\delta^{15}N_{enamel}$ values. Analogously, Wißing et al.[30] found that Pleistocene cave hyenas have higher $\delta^{15}N_{collagen}$ values than coexisting carnivore species, however this enrichment has not been documented in $\delta^{15}N$ of modern spotted hyenas and lions in Africa (i.e., measured in hair and feces; Codron et al.[31]), and it is well-known that both predator species exhibit a large overlap in prey choice (Hayward et al.[32]). While the possibility that bone consumption have contributed to the elevated $\delta^{15}N_{enamel}$ values observed in the spotted hyenas cannot be entirely ruled out, this explanation seems unlikely. This is because the primary source of nitrogen in bones should come from either bone collagen (which was measured and is not especially elevated in $\delta^{15}N$ compared to other tissues) or bone marrow. There is very little data on bone marrow $\delta^{15}N$ values in the literature, but the values that do exist are not especially elevated in $\delta^{15}N$ either (Drucker and Bocherens[33]). Therefore, unless considerable fractionation occurs during the metabolism of collagen in hyena digestive system, it is unlikely that this is driving the higher $\delta^{15}N_{enamel}$ values observed in this taxon. A more likely explanation is the early the tooth mineralization (11 to 13 months) relative to tooth eruption schedule (between 12 and 14 months) in spotted hyenas resulting in a nursing signal in our $\delta^{15}N_{enamel}$ values.

Variation in $\delta^{13}C$ values in tooth enamel

We measured the carbon isotope composition of tooth enamel bioapatite (i.e., structurally bound carbonate; $\delta^{13}C_{enamel}$) in the same aliquot of tooth enamel powder used to measure $\delta^{15}N_{enamel}$ using the 'cold trap method' of Vonhof et al.[34], which permits high precision analysis of very small (50–100 µg) amounts of enamel. Carbon isotopes are widely used to reconstruct the type of vegetation (C_3 *versus* C_4 plants) consumed by animals (and subsequently by the

predators that feed on them)[35-40]. The ability to measure paired carbon and nitrogen isotopic values in a single aliquot of tooth enamel is an important step forward for paleodietary studies.

Herbivores:

As anticipated, browsing and grazing herbivores are separable by their $\delta^{13}C_{enamel}$ values, reflecting the consumption of C_3 and C_4 plants, respectively, with mixed feeders falling in between. Gorillas (*Gorilla gorilla*) have the lowest $\delta^{13}C_{enamel}$ values, along with duikers (*Philantomba monticola*) and the black rhino (*Diceros bicornis*), reflecting predominantly C_3 consumption and possibly a small canopy effect for the gorillas and duikers[41]. One grazer, the hippopotamus (*Hippopotamus amphibius*) has a $\delta^{13}C$ value typical for a mixed feeder, likely due to the consumption of C_3 grasses which grow near water sources. For mixed feeders, $\delta^{13}C_{enamel}$ values indicate a predominantly C_3-based diet for elephants (*Loxodonta africana*) and springbok (*Antidorcas marsupialis*), and a more C_4-based diet for the impala (*Aepyceros melampus*).

Omnivores:

Existing carbon isotope data indicates that, overall, the typical baboon diet is comprised predominantly of C_3 foods and some C_4 foods (but that the consumption of C_4-based foods is quite variable between environments), thus $\delta^{13}C_{enamel}$ values for baboons (*Papio*) in this study are consistent with previously published data.

Carnivores:

Isotopic spacing in $\delta^{13}C$ between enamel and collagen (i.e., $\Delta^{13}C_{enamel\text{-}bone\ collagen}$) was, as expected, greater in herbivores compared to carnivores. An offset between the two tissue types occurs because $\delta^{13}C_{collagen}$ reflects the protein component of diet only, whereas $\delta^{13}C_{enamel}$ reflects the whole diet (e.g., protein, carbohydrates, and fats[42]). Thus carnivores, which consume a protein-rich diet, have lower $\Delta^{13}C_{enamel\text{-}bone\ collagen}$ values (4.3 to 4.8‰) compared to herbivores (6.8 to 7.6‰), whose plant-based diet typically contains less crude protein and greater proportions of carbohydrates[43,44]. The $\Delta^{13}C_{enamel\text{-}bone\ collagen}$ values we observed in this study are therefore in line with the offsets documented in the literature.

The four THM fossil specimens for which $\delta^{13}C$ enamel and collagen values were obtained are also plotted in Fig. S4. The $\delta^{13}C$ values of the THM fossils have been corrected (by -0.7‰) to account for shifts in the $\delta^{13}C$ of atmospheric CO_2[45,46] and to permit direct comparison with the African mammals which were collected between 1950 and 1970. The THM fossils exhibit a $\Delta^{13}C_{enamel\text{-}bone\ collagen}$ that is consistent with that of the modern data. All three herbivores fall along the herbivore regression line as does the single omnivore (pig), suggesting a predominantly herbivorous diet.

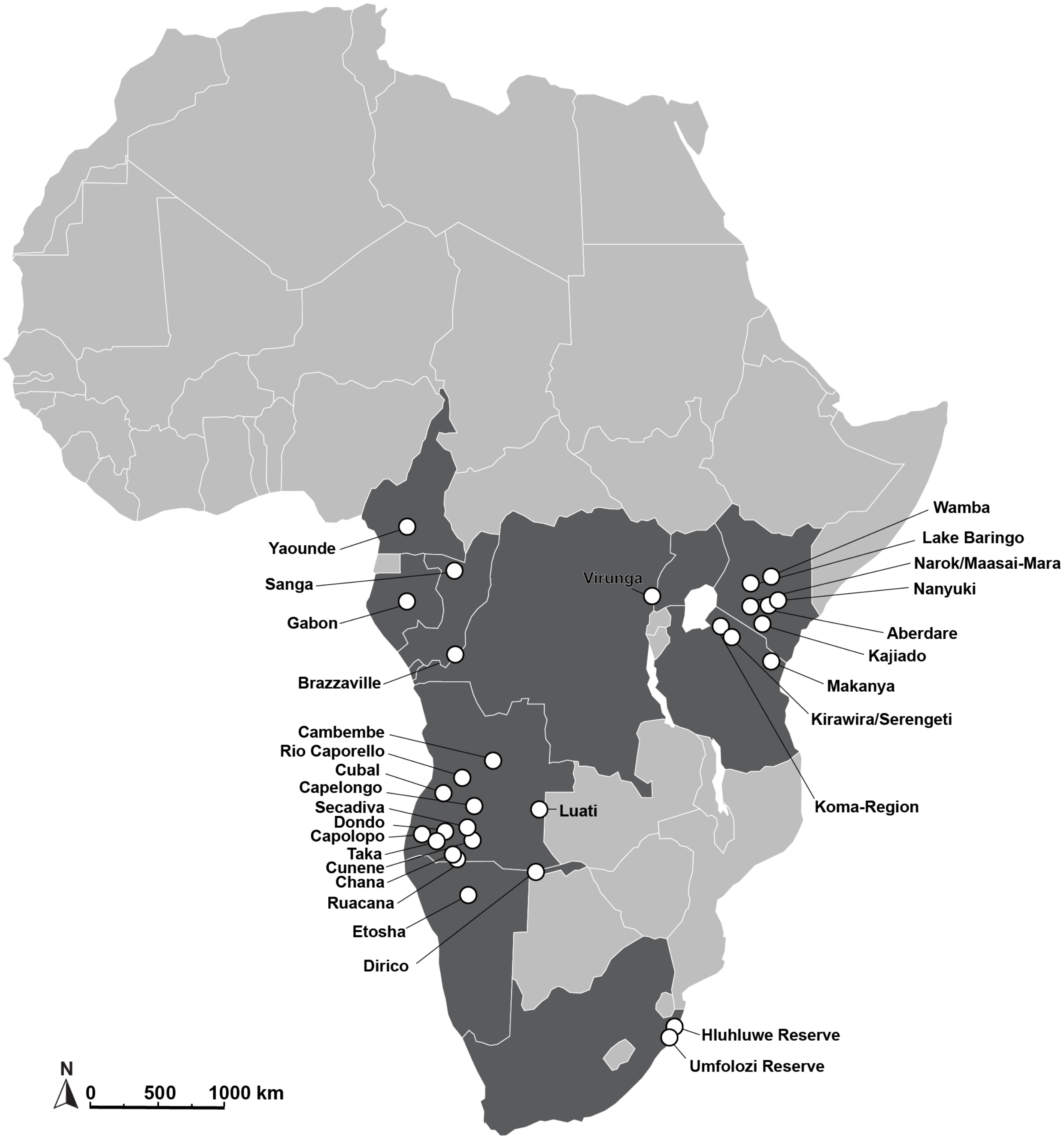

Fig. S1. Map of Africa with sampling localities for modern mammals analyzed for their enamel and bone collagen nitrogen isotope composition. For information about the localities (vegetation, climate, altitude), see Table S1.

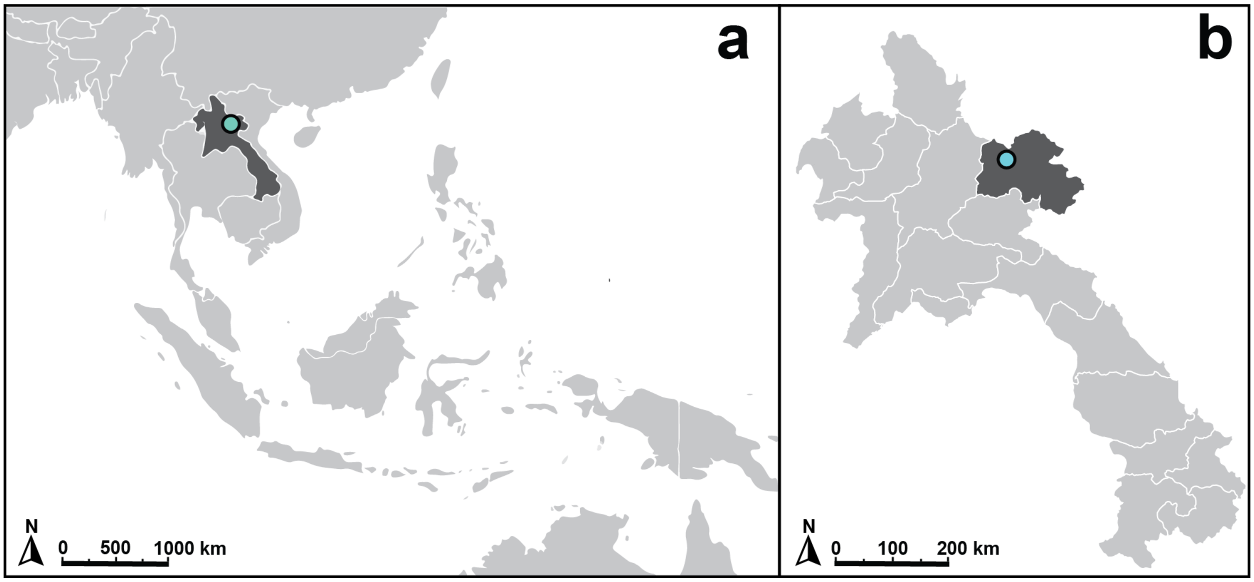

Fig. S2. Map indicating location of Tam Hay Marklot (THM) in southeast Asia (a) and in the Hua Pan Province in Laos (b). This cave locality yielded the Late Pleistocene mammalian teeth analyzed in this study. For further information about geological context, sedimentary deposits, cave filling history, faunal description, chronology, ecology and dating, see Bourgon et al.[47].

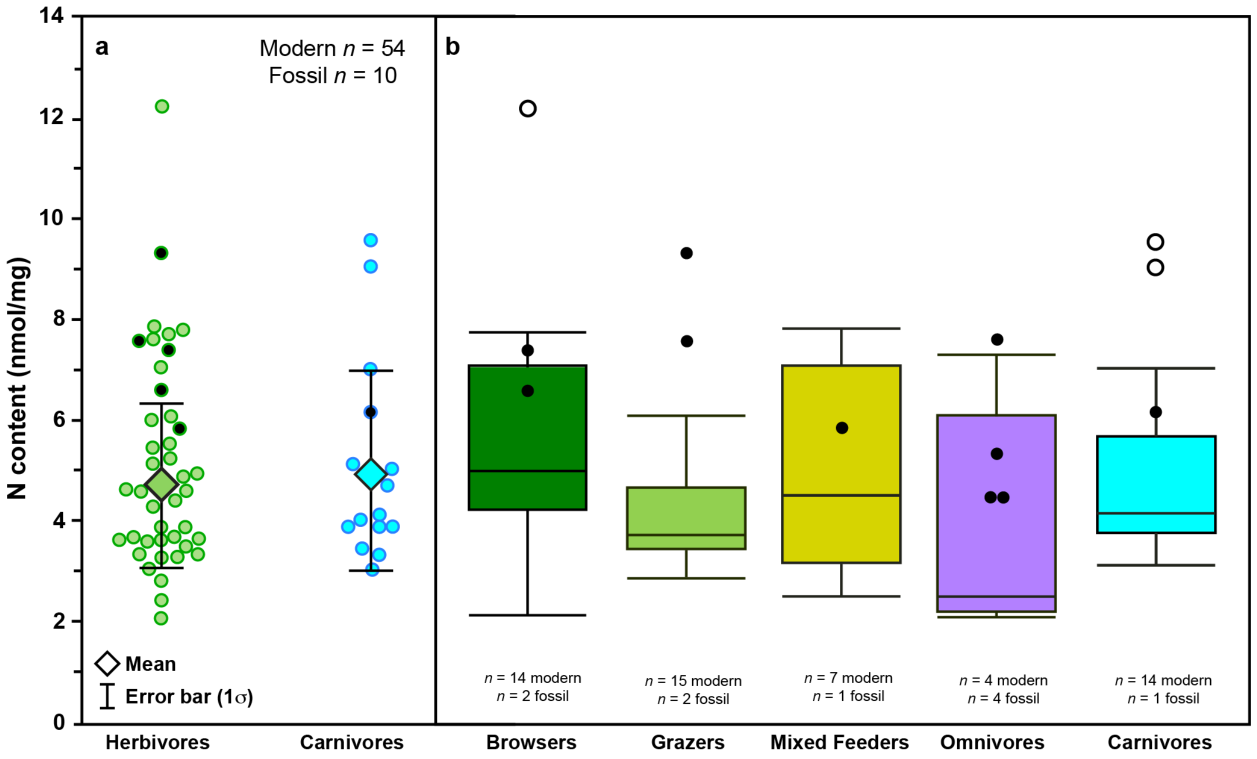

Fig. S3. Nitrogen contents for all enamel samples analyzed in this study. Colored circles (a) and box plots (b) refer to modern African mammal data, filled black circles indicate fossil THM specimens (a and b). Fossil data is excluded from calculations for box plots, means, and standard deviations. Open white circles denote outliers in the modern faunal dataset. Modern and fossil tooth enamel N contents fall in the same range and no differences in N content were observed between dietary groups. Data presented as box plots with median and 25th percentiles indicated. Individual data points for modern fauna for panel b are provided in Table 1 and Supplementary Data 1.

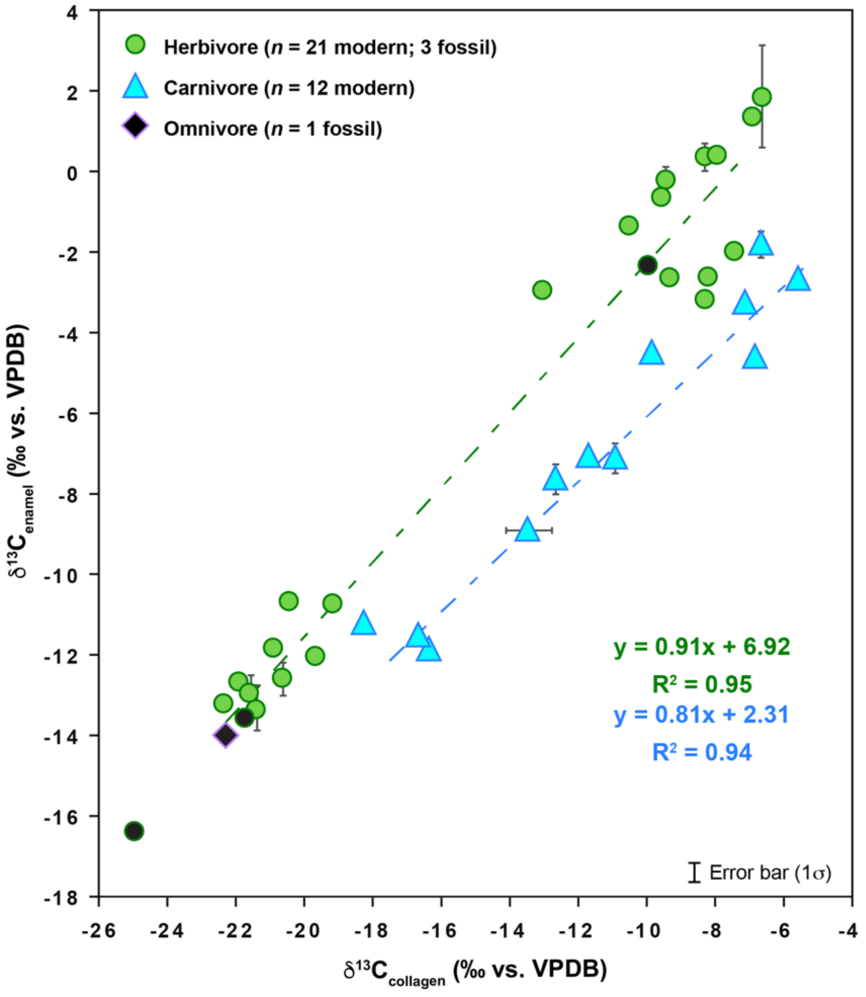

Fig. S4. Regression of paired $\delta^{13}C_{enamel}$ *versus* $\delta^{13}C_{collagen}$ values ($\bar{x} \pm 1\sigma$) for all modern African mammals (colored symbols; *n* = 33) and THM fossils (black symbols, *n* = 4; $\delta^{13}C_{dentin\text{-}collagen}$). The dashed lines and equations indicate the regression between the two variables for modern herbivores (green) and carnivores (blue). $\delta^{13}C$ values of the THM fossils have been corrected (by -0.7 ‰) to account for shifts in the $\delta^{13}C$ of atmospheric CO_2[45,46] and to permit direct comparison with the African mammals which were collected between 1950 and 1970. Note the positive correlation between bone collagen and enamel values within each diet group, as well as the higher $\delta^{13}C_{bone\text{-}collagen}$ values for the carnivores compared to the herbivores.

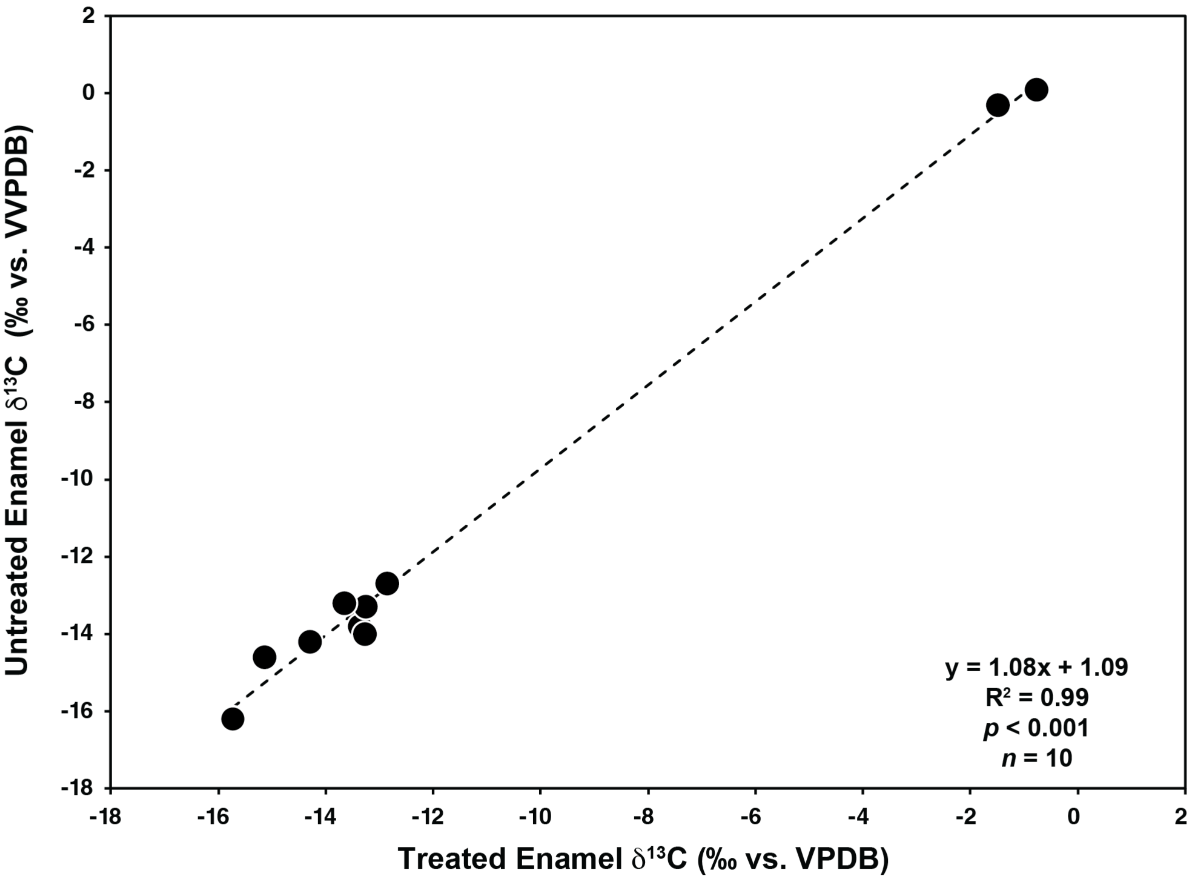

Fig. S5: Regression analysis of untreated *versus* treated $\delta^{13}C_{enamel}$ values of matched samples. The values for the ten untreated fossil enamel samples included in this study were compared to values obtained from the same teeth, but from different sample aliquots that were pre-treated with 0.1 M acetic acid for four hours as reported in Bourgon et al.[47]. Comparison of matched treated *versus* untreated enamel show a significant and near perfect correlation.

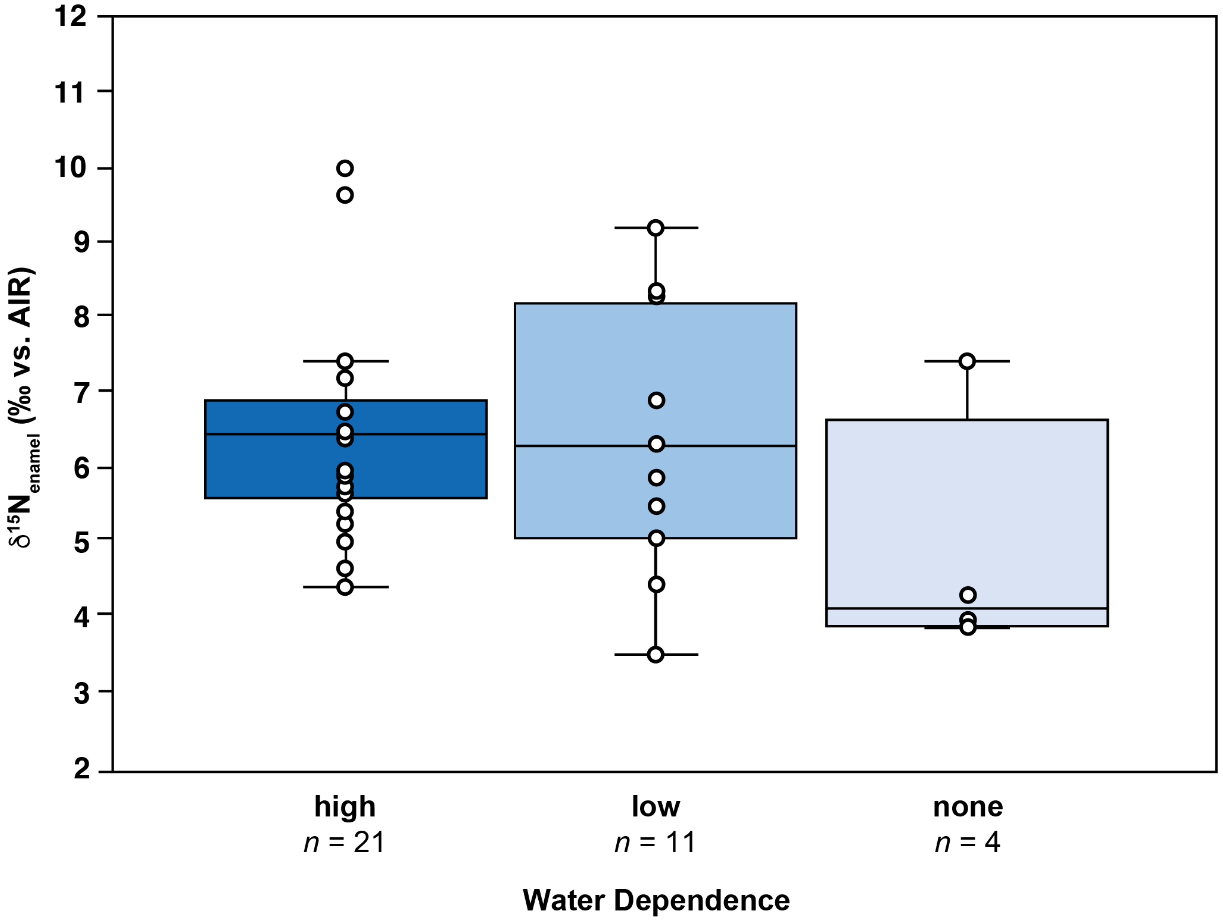

Fig. S6. δ^{15}N$_{enamel}$ values for modern African herbivores grouped according to their water dependency in decreasing order (categories after[29,48-50]). Herbivores do not show statistically significant differences ($\chi^2(2) = 3$, $p = 0.233$; see Table S4 for pairwise comparisons) according to water dependence. Data presented as box plots with median and 25th percentiles indicated, open white circles represent individual data points.

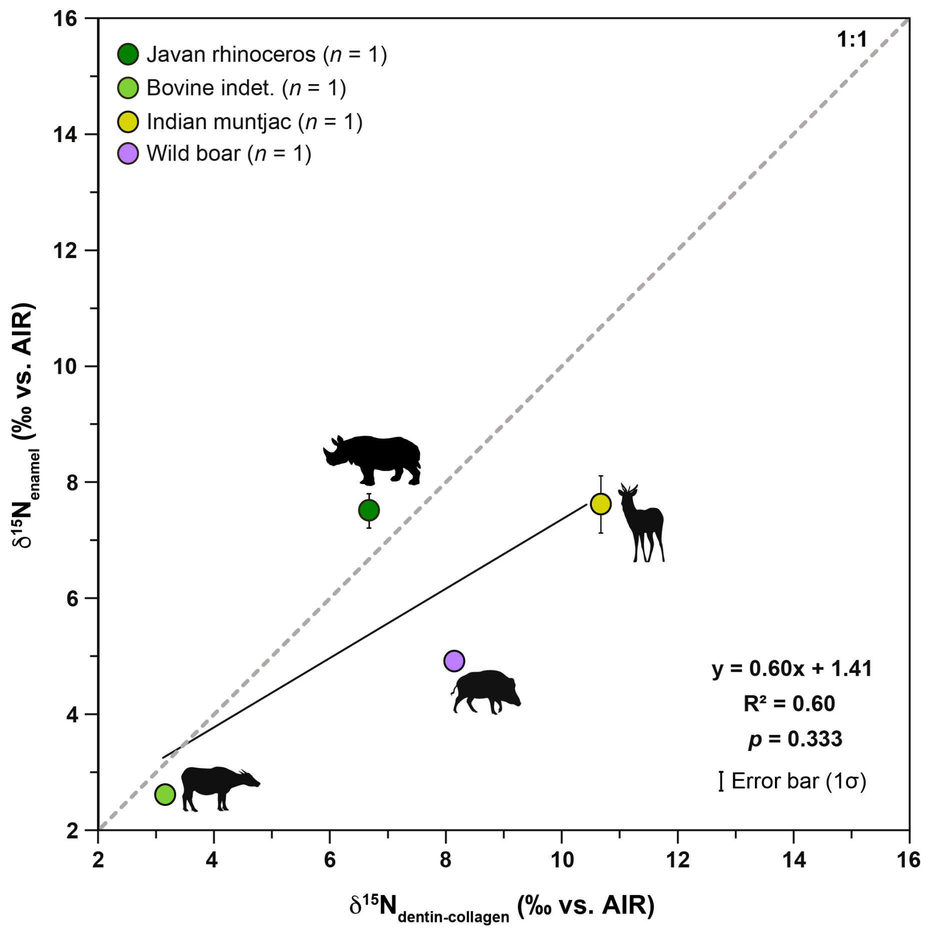

Fig. S7. Regression of paired $\delta^{15}N_{enamel}$ *versus* $\delta^{15}N_{dentin\text{-}collagen}$ values ($\bar{x} \pm 1\sigma$) for the fossil Tam Hay Marklot specimens (*n* = 4). The dashed line represents a 1:1 correlation between dentin collagen and enamel values. The solid line indicates the true regression. A positive relationship between measured $\delta^{15}N_{dentin\text{-}collagen}$ and $\delta^{15}N_{enamel}$ is present (see also Fig. 2).

Table S1: Table with modern African mammal locality information (see Figure S1 for map), including altitude in meters above sea level, mean annual precipitation (MAP) in mm/year, mean annual temperature (MAT) in °C (data from WorldClim Version 2.1 bioclimatic variables[51] and dominate vegetation type[52-56].

Country	Locality	Altitude (m)	MAP (mm/year)	MAT (°C)	Dominant vegetation type
**Angola**	Cambembe	1127	1204	21	Angolan Miombo Woodlands
**Angola**	Capelongo	1449	968	21	Angolan Miombo Woodlands
**Angola**	Capolopoppo	526	213	24	Namibian Savanna Woodlands
**Angola**	Chana	1183	494	22	Angolan Mopane Woodlands, Zambezian Baikiaea Woodlands
**Angola**	Cubal	919	957	22	Angolan Scarp Savanna and Woodlands
**Angola**	Cunene	1425	674	23	Angolan Mopane Woodlands, Zambezian Baikiaea Woodlands
**Angola**	Dirico	1239	651	22	Zambezian Baikiaea Woodlands
**Angola**	Dondo	73	701	25	Angolan miombo woodlands and Angolan mopane woodlands
**Angola**	Luati	1196	933	20	Zambezian Baikiaea Woodlands: Brachystegia, Berlinia, Monotes
**Angola**	Rio Caporello	1695	1390	20	Angolan Miombo woodlands
**Angola**	Ruacana	865	338	23	Savanna with thorn trees and broad deciduous trees (Combretaceae), often forest-like, also *Copaifera mopane*
**Angola**	Secadiva	1591	840	20	Angolan miombo woodlands and Angolan mopane woodlands
**Angola**	Taka Region	1173	502	21	Angolan miombo woodlands and Angolan mopane woodlands
**Cameroon**	Yaoundé	722	1604	24	Submontane (900-1500 m) and Montane (>1500 m) forest
**Congo**	Brazzaville	389	1482	25	Humid tropical forest
**Congo**	Sanga	678	1614	24	Rain forest, riverine forest, swamp forest, monospecific forest of Limbali (*Gilbertiodendron dewevrei*)
**Gabon**	Gabon	118	2079	23	Montane forest
**Kenya**	Aberdare	3419	1491	9	Mountain rainforest
**Kenya**	Kajiado	1734	554	19	Savanna with thorn trees
**Kenya**	Lake Baringo	983	659	23	Acacia and Commiphora species with little undergrowth
**Kenya**	Nanyuki	1944	851	16	Savanna with thorn trees
**Kenya**	Narok/Maasai-Mara	1852	784	17	Savanna with thorn trees
**Kenya**	Wamba	1297	795	20	Savanna with thorn trees
**Namibia**	Etosha Pan	1186	422	23	Dry savanna with broad deciduous trees (Combretaceae), often forest-like, also *Copaifera mopane*
**South Africa**	Hluhluwe Reservation	348	1031	20	Savanna with thorn trees, subtropical rainforest
**South Africa**	Umfolozi Reservation	138	969	21	Savanna with thorn trees, subtropical rainforest
**Tanzania**	Kirawira/Serengeti	1469	842	21	Savanna with thorn and broad-leaved trees
**Tanzania**	Koma, Mara Region	1134	1043	21	Wet tropical savanna
**Tanzania**	Makania National Park	824	693	23	Grasslands and woody savanna mosaic
**Uganda**	Virunga National Park	1278	1239	21	Evergreen tropical rainforest

Table S2: Results of post-hoc Tukey-Howell pairwise comparisons of $\delta^{15}N$ values from enamel bound organic matter and bone collagen for modern African mammals.

Pair	Difference	Lower CI	Upper CI	*p*-value	Significant
			$\delta^{15}N_{enamel}$		
Browser – Grazer	0.273	-1.388	1.934	0.990	NS
Browser – Mixed Feeder	1.743	-0.326	3.812	0.137	NS
Browser – Omnivore	1.625	-0.909	4.159	0.376	NS
Browser – Carnivore	4.129	2.439	5.818	< 0.001	*
Grazer – Mixed Feeder	1.470	-0.576	3.516	0.266	NS
Grazer – Omnivore	1.352	-1.164	3.867	0.554	NS
Grazer – Carnivore	3.855	2.194	5.516	< 0.001	*
Mixed Feeder – Omnivore	0.118	-2.684	2.919	1.000	NS
Mixed Feeder – Carnivore	2.386	0.317	4.455	0.016	*
Omnivore – Carnivore	2.504	-0.031	5.038	0.054	NS
			$\delta^{15}N_{bone\text{-}collagen}$		
Browser – Grazer	0.319	-1.978	2.616	0.981	NS
Browser – Mixed Feeder	3.228	-0.020	6.477	0.052	NS
Browser – Carnivore	3.792	1.495	6.089	0.001	*
Grazer – Mixed Feeder	2.909	-0.056	5.874	0.056	NS
Grazer – Carnivore	3.473	1.597	5.348	0.000	*
Mixed Feeder – Carnivore	0.563	-2.402	3.529	0.954	NS

Table S3: Results of pairwise comparisons of $\delta^{13}C$ values of enamel bioapatite and bone collagen using Dunn's post-hoc test with a Bonferroni correction for all measured modern African mammals.

Pair	Mean Rank Difference	Z	SE	*p*-value	Significant
		**$\delta^{13}C_{enamel}$**			
Browser – Grazer	-36.910	6.315	5.845	< 0.001	*
Browser – Mixed Feeder	-13.857	1.903	7.281	0.057	NS
Browser – Omnivore	-15.643	1.754	8.917	0.079	NS
Browser – Carnivore	-20.964	3.527	5.945	0.000	*
Grazer – Mixed Feeder	23.052	3.202	7.199	0.001	*
Grazer – Omnivore	21.267	2.403	8.851	0.016	NS
Grazer – Carnivore	15.945	2.728	5.845	0.006	NS
Mixed Feeder – Omnivore	-1.786	0.181	9.858	0.856	NS
Mixed Feeder – Carnivore	-7.107	0.976	7.281	0.329	NS
Omnivore – Carnivore	-5.321	0.597	8.917	0.551	NS
			**$\delta^{13}C_{bone-collagen}$**		
Browser – Grazer	-18.917	3.914	4.834	< 0.001	*
Browser – Mixed Feeder	-1.500	0.219	6.836	0.826	NS
Browser – Carnivore	-15.083	3.121	4.834	0.002	*
Grazer – Mixed Feeder	17.417	2.791	6.240	0.005	*
Grazer – Carnivore	3.833	0.971	3.947	0.331	NS
Mixed Feeder – Carnivore	-13.583	2.177	6.240	0.030	NS

Table S4: Results of pairwise comparisons of $\delta^{15}N$ values of enamel and bone collagen according to water dependency (categories high, low, none after[29,48-50]) using Dunn's post-hoc test with a Bonferroni correction for all measured modern African mammals.

Pair	Mean Rank Difference	Z	SE	*p*-value	Significant
		**$\delta^{15}N_{enamel}$**			
High – Low	0.9957	0.2542	3.9173	0.7994	NS
High – None	9.9048	1.725	5.7417	0.0845	NS
Low – None	8.9091	1.4498	6.1452	0.1471	NS
			**$\delta^{15}N_{bone-collagen}$**		
High – Low	2.117	0.641	3.301	0.521	NS
High – None	4.792	1.338	3.580	0.181	NS
Low – None	2.675	0.643	4.160	0.520	NS

Supplementary References

1 Fuller, B. T., Fuller, J. L., Harris, D. A. & Hedges, R. E. M. Detection of breastfeeding and weaning in modern human infants with carbon and nitrogen stable isotope ratios. *American Journal of Physical Anthropology* **129**, 279-293, doi:https://doi.org/10.1002/ajpa.20249 (2006).

2 Tsutaya, T. & Yoneda, M. Reconstruction of breastfeeding and weaning practices using stable isotope and trace element analyses: A review. *American journal of physical anthropology* **156 Suppl 59**, 2-21, doi:10.1002/ajpa.22657 (2015).

3 Dailey-Chwalibóg, T. *et al.* Weaning and stunting affect nitrogen and carbon stable isotope natural abundances in the hair of young children. *Scientific Reports* **10**, 2522, doi:10.1038/s41598-020-59402-8 (2020).

4 Chinique de Armas, Y., Mavridou, A.-M., Garcell Domínguez, J., Hanson, K. & Laffoon, J. Tracking breastfeeding and weaning practices in ancient populations by combining carbon, nitrogen and oxygen stable isotopes from multiple non-adult tissues. *PloS one* **17**, e0262435, doi:10.1371/journal.pone.0262435 (2022).

5 Stansfield, F. J. A Novel Objective Method of Estimating the Age of Mandibles from African Elephants (Loxodonta africana Africana). *PloS one* **10**, e0124980, doi:10.1371/journal.pone.0124980 (2015).

6 Lee, P. C. & Cynthia, J. M. Early Maternal Investment in Male and Female African Elephant Calves. *Behav. Ecol. Sociobiol.* **18**, 353-361 (1986).

7 Smuts, G. L., Anderson, J. L. & Austin, J. C. Age determination of the African lion (*Panthera leo*). *Journal of Zoology* **185**, 115-146 (1978).

8 White, P. A. *et al.* Age Estimation of African Lions Panthera leo by Ratio of Tooth Areas. *PloS one* **11**, e0153648, doi:10.1371/journal.pone.0153648 (2016).

9 Hillson, S. *Teeth*. 2 edn, (Cambridge University Press, 2005).

10 Stander, P. E. Field age determination of leopards by tooth wear. *African Journal of Ecology* **35**, 156-161 (1997).

11 Gittleman, J. L. Carnivore Life History Patterns: Allometric, Phylogenetic, and Ecological Associations. *The American Naturalist* **127**, 744-771, doi:10.1086/284523 (1986).

12 Binder, W. J. & Van Valkenburgh, B. Development of bite strength and feeding behaviour in juvenile spotted hyenas (Crocuta crocuta). *Journal of Zoology* **252**, 273-283, doi:10.1111/j.1469-7998.2000.tb00622.x (2000).

13 Murphy, B. P. & Bowman, D. M. J. S. Kangaroo metabolism does not cause the relationship between bone collagen $\delta^{15}N$ and water availability. *Functional Ecology* **20**, 1062-1069, doi:10.1111/J.1365-2435.2006.01186.X (2006).

14 Minagawa, M. & Wada, E. Stepwise enrichment of ^{15}N along food chains: further evidence and the relation between ^{15}N and animal age. *Geochimica et Cosmochimica Acta* **48**, 1135-1140 (1984).

15 Schoeninger, M. J. Stable Isotope Analyses and the Evolution of Human Diets. *Annual Review of Anthropology* **43**, 413-430, doi:10.1146/annurev-anthro-102313-025935 (2014).

16 Ambrose, S. H. Stable carbon and nitrogen isotope analysis of human and animal diet in Africa. *Journal of Human Evolution* **15**, 707-731 (1986).

17 Ambrose, S. H. Effects of diet, climate and physiology on nitrogen isotope abundances in terrestrial foodwebs. *Journal of Archaeological Science* **18**, 293-317, doi:10.1016/0305-4403(91)90067-Y (1991).
18 Austin, A. T. & Vitousek, P. M. Nutrient dynamics on a precipitation gradient in Hawai'i. *Oecologia* **113**, 519-529, doi:10.1007/s004420050405 (1998).
19 Amundson, R. *et al.* Global patterns of the isotopic composition of soil and plant nitrogen. *Global Biogeochemical Cycles* **17**, 31-31 - 31-10, doi:10.1029/2002gb001903 (2003).
20 Swap, R. Natural abundance of ^{13}C and ^{15}N in C_3 and C_4 vegetation of southern Africa: patterns and implications. *Global Change Biology* **10**, 350-358, doi:10.1046/j.1529-8817.2003.00702.x (2004).
21 Handley, L. *et al.* The ^{15}N natural abundance ($\delta^{15}N$) of ecosystem samples reflects measures of water availability. *Functional Plant Biology* **26**, 185-199 (1999).
22 Ambrose, S. H. & DeNiro, M. J. The isotopic ecology of East-African mammals. *Oecologia* **69**, 395-406, doi:10.1007/bf00377062 (1986).
23 Sealy, J. C., van der Merwe, N. J., Thorp, J. A. L. & Lanham, J. L. Nitrogen isotopic ecology in southern Africa: Implications for environmental and dietary tracing. *Geochimica et Cosmochimica Acta* **51**, 2707-2717, doi:10.1016/0016-7037(87)90151-7 (1987).
24 Heaton, T. H. E., Vogel, J. C., Von La Chevallerie, G. & Collett, G. Climatic influence on the isotopic composition of bone nitrogen. *Nature* **322**, 822-823, doi:10.1038/322822a0 (1986).
25 Ambrose, S. H. & DeNiro, M. J. Reconstruction of African human diet using bone collagen carbon and nitrogen isotope ratios. *Nature* **319**, 321-324, doi:10.1038/319321a0 (1986).
26 Schwarcz, H. P., Dupras, T. L. & Fairgrieve, S. I. ^{15}N Enrichment in the Sahara: In Search of a Global Relationship. *Journal of Archaeological Science* **26**, 629-636, doi:10.1006/JASC.1998.0380 (1999).
27 Koch, P. in *Stable Isotopes in Ecology and Environmental Science, Second Edition* (eds Robert Michener & Kate Lajtha) 99-154 (Blackwell Publishing Ltd, 2007).
28 Balter, V., Simon, L., Fouillet, H. & Lécuyer, C. Box-modeling of $^{15}N/^{14}N$ in mammals. *Oecologia* **147**, 212-222, doi:10.1007/s00442-005-0263-5 (2006).
29 Hempson, G., Archibald, S. & Bond, W. A continent-wide assessment of the form and intensity of large mammal herbivory in Africa. *Science* **350**, 1056-1061, doi:10.1126/science.aac7978 (2015).
30 Wißing, C. *et al.* Stable isotopes reveal patterns of diet and mobility in the last Neandertals and first modern humans in Europe. *Scientific Reports* **9**, 1-12, doi:10.1038/s41598-019-41033-3 (2019).
31 Codron, D., Codron, J., Sponheimer, M. & Clauss, M. Within-population isotopic niche variability in savanna mammals: Disparity between carnivores and herbivores. *Frontiers in Ecology and Evolution* **4**, doi:10.3389/fevo.2016.00015 (2016).
32 Hayward, M. W. Prey preferences of the spotted hyaena (*Crocuta crocuta*) and degree of dietary overlap with the lion (*Panthera leo*). *Journal of Zoology* **270**, 606-614, doi:10.1111/J.1469-7998.2006.00183.X (2006).

33 Drucker, D. & Bocherens, H. Carbon and nitrogen stable isotopes as tracers of change in diet breadth during Middle and Upper Palaeolithic in Europe. *International Journal of Osteoarchaeology* **14**, 162-177, doi:https://doi.org/10.1002/oa.753 (2004).
34 Vonhof, H. *et al.* High-precision stable isotope analysis of <5 μg $CaCO_3$ samples by continuous-flow mass spectrometry. *Rapid Communications in Mass Spectrometry* **34**, e8878, doi:10.1002/rcm.8878 (2020).
35 Cerling, T. E. & Harris, J. Carbon isotope fractionation betwwen diet and bioapatite in ungulate mammals and implications for ecological and palaecological studies. *Oecologia* **120**, 347-363 (1999).
36 Cerling, T. E., Harris, J. M. & Passey, B. H. Diets of East African Bovidae based on stable isotope analysis. *Journal of Mammalogy* **84**, 456-470, doi:10.1644/1545-1542(2003)084<0456:DOEABB>2.0.CO;2 (2003).
37 Kohn, M. J. & Cerling, T. E. Stable isotope compositions of biological apatite. *Phosphates: Geochemical, Geobiological and Materials Importance* **48**, 455-488, doi:10.2138/rmg.2002.48.12 (2019).
38 Uno, K. T. *et al.* Large mammal diets and paleoecology across the Oldowan–Acheulean transition at Olduvai Gorge, Tanzania from stable isotope and tooth wear analyses. *Journal of Human Evolution* **120**, 76-91, doi:10.1016/j.jhevol.2018.01.002 (2018).
39 Lüdecke, T. and Leichliter, J. *et al.* Carbon, nitrogen, and oxygen stable isotopes in modern tooth enamel: A case study from Gorongosa National Park, central Mozambique. *Frontiers in Ecology and Evolution* **10**, doi:10.3389/fevo.2022.958032 (2022).
40 Hopley, P. J. *et al.* Stable isotope analysis of carnivores from the Turkana Basin, Kenya: Evidence for temporally-mixed fossil assemblages. *Quaternary International*, doi:https://doi.org/10.1016/j.quaint.2022.04.004 (2022).
41 van der Merwe, N. J. & Medina, E. The canopy effect, carbon isotope ratios and foodwebs in amazonia. *Journal of Archaeological Science* **18**, 249-259, doi:https://doi.org/10.1016/0305-4403(91)90064-V (1991).
42 Ambrose, S. H. & Norr, L. in *Prehistoric Human Bone: Archaeology at the Molecular Level* (eds Joseph B. Lambert & Gisela Grupe) 1-37 (Springer Berlin Heidelberg, 1993).
43 Lee-Thorp, J. A., Sealy, J. C. & van der Merwe, N. J. Stable carbon isotope ratio differences between bone collagen and bone apatite, and their relationship to diet. *Journal of Archaeological Science* **16**, 585-599, doi:10.1016/0305-4403(89)90024-1 (1989).
44 Clementz, M., Fox-Dobbs, K., Wheatley, P. V., Koch, P. & Doak, D. F. Revisiting old bones: coupled carbon isotope analysis of bioapatite and collagen as an ecological and palaeoecological tool. *Geological Journal* **44**, 605-620, doi:10.1002/gj.1173 (2009).
45 Hare, V. J., Loftus, E., Jeffrey, A. & Ramsey, C. B. Atmospheric CO_2 effect on stable carbon isotope composition of terrestrial fossil archives. *Nature communications* **9**, 1-8 (2018).
46 Dombrosky, J. A ~1000-year 13C Suess correction model for the study of past ecosystems. *The Holocene* **30**, 474-478, doi:10.1177/0959683619887416 (2020).
47 Bourgon, N. *et al.* Zinc isotopes in Late Pleistocene fossil teeth from a Southeast Asian cave setting preserve paleodietary information. *Proceedings of the National Academy of Sciences of the United States of America* **117**, 4675-4681, doi:10.1073/pnas.1911744117 (2020).

48 Hamilton, W. J. Namib Desert chacma baboon (*Papio ursinus*) use of food and water resources during a food shortage. *Madoqua* **1986**, 397-407 (1986).
49 Bothma, J. d. P. Water-use by southern Kalahari leopards. *South African Journal of Wildlife Research* **35**, 131-137 (2005).
50 Wilson, D. E. & Mittermeier, R. A. *Handbook of the Mammals of the World*. Vol. 1 (Lynx Wsicions, 2009).
51 Fick, S. E. & Hijmans, R. J. WorldClim 2: new 1-km spatial resolution climate surfaces for global land areas. *International Journal of Climatology* **37**, 4302-4315, doi:10.1002/joc.5086 (2017).
52 Mendelsohn, J., Jarvis, A., C, R. & Robertson, T. *Atlas of Namibia: A Portrait of the Land and its People*. (2002).
53 Kiage, L. & Liu, K.-b. Paleoenvironmental Changes in the Lake Baringo Basin, Kenya, East Africa Since AD 1650: Evidence from the Paleorecord∗. *The Professional Geographer* **61**, 438-458, doi:10.1080/00330120903143425 (2009).
54 Martin, E. H. *et al.* in *Protected Areas in Northern Tanzania: Local Communities, Land Use Change, and Management Challenges* (eds Jeffrey O. Durrant *et al.*) 145-155 (Springer International Publishing, 2020).
55 Le Houérou, H. N. *Bioclimatology and Biogeography of Africa*. (Springer Verlag, 2009).
56 Landesaufnahme, R. f. *Afrika-Handbuch Band VI, Pflanzengeographie Afrikas*. (Reichsamt für Landesaufnahme, 1963).
