# Supplementary material for: Tooth enamel nitrogen isotope composition records trophic position: a tool for reconstructing food webs
Source: Commun Biol. 2023 Apr 7;6:373. doi: 10.1038/s42003-023-04744-y (PMC10082005; doi:10.1038/s42003-023-04744-y)
Supplement: Supplementary file 3 — Description of Additional Supplementary Files [file 42003_2023_4744_MOESM3_ESM.pdf]

Description of Additional Supplementary Files

File name: Supplementary Data 1-4
Description: : Isotopic data for all analyzed samples and standards.
