# Supplementary material for: Tooth enamel nitrogen isotope composition records trophic position: a tool for reconstructing food webs
Source: Commun Biol. 2023 Apr 7;6:373. doi: 10.1038/s42003-023-04744-y (PMC10082005; doi:10.1038/s42003-023-04744-y)
Supplement: Supplementary file 5 — Reporting Summary [file 42003_2023_4744_MOESM5_ESM.pdf]

nature portfolio

Corresponding author(s): Jennifer N. Leichliter
Tina Lüdecke

Last updated by author(s): Mar 17, 2023

Reporting Summary

Nature Portfolio wishes to improve the reproducibility of the work that we publish. This form provides structure for consistency and transparency in reporting. For further information on Nature Portfolio policies, see our Editorial Policies and the Editorial Policy Checklist.

Statistics

For all statistical analyses, confirm that the following items are present in the figure legend, table legend, main text, or Methods section.

n/a	Confirmed	
☐	☒	The exact sample size (*n*) for each experimental group/condition, given as a discrete number and unit of measurement
☐	☒	A statement on whether measurements were taken from distinct samples or whether the same sample was measured repeatedly
☐	☒	The statistical test(s) used AND whether they are one- or two-sided<br>*Only common tests should be described solely by name; describe more complex techniques in the Methods section.*
☐	☒	A description of all covariates tested
☐	☒	A description of any assumptions or corrections, such as tests of normality and adjustment for multiple comparisons
☐	☒	A full description of the statistical parameters including central tendency (e.g. means) or other basic estimates (e.g. regression coefficient) AND variation (e.g. standard deviation) or associated estimates of uncertainty (e.g. confidence intervals)
☐	☒	For null hypothesis testing, the test statistic (e.g. *F*, *t*, *r*) with confidence intervals, effect sizes, degrees of freedom and *P* value noted<br>*Give P values as exact values whenever suitable.*
☒	☐	For Bayesian analysis, information on the choice of priors and Markov chain Monte Carlo settings
☒	☐	For hierarchical and complex designs, identification of the appropriate level for tests and full reporting of outcomes
☐	☒	Estimates of effect sizes (e.g. Cohen's *d*, Pearson's *r*), indicating how they were calculated

Our web collection on statistics for biologists contains articles on many of the points above.

Software and code

Policy information about availability of computer code

Data collection: *Provide a description of all commercial, open source and custom code used to collect the data in this study, specifying the version used OR state that no software was used.*

Data analysis: *Provide a description of all commercial, open source and custom code used to analyse the data in this study, specifying the version used OR state that no software was used.*

For manuscripts utilizing custom algorithms or software that are central to the research but not yet described in published literature, software must be made available to editors and reviewers. We strongly encourage code deposition in a community repository (e.g. GitHub). See the Nature Portfolio guidelines for submitting code & software for further information.

Data

Policy information about availability of data

All manuscripts must include a data availability statement. This statement should provide the following information, where applicable:

- Accession codes, unique identifiers, or web links for publicly available datasets
- A description of any restrictions on data availability
- For clinical datasets or third party data, please ensure that the statement adheres to our policy

The Supplementary Materials includes additional information regarding analytical procedures, data processing, sampling locations, and isotopic data for the samples included in this study. All isotope values are provided in the main text as well and in an associated excel database (Supplementary Data 1–4).

Human research participants

Policy information about studies involving human research participants and Sex and Gender in Research.

Reporting on sex and gender	*Use the terms sex (biological attribute) and gender (shaped by social and cultural circumstances) carefully in order to avoid confusing both terms. Indicate if findings apply to only one sex or gender; describe whether sex and gender were considered in study design whether sex and/or gender was determined based on self-reporting or assigned and methods used. Provide in the source data disaggregated sex and gender data where this information has been collected, and consent has been obtained for sharing of individual-level data; provide overall numbers in this Reporting Summary. Please state if this information has not been collected. Report sex- and gender-based analyses where performed, justify reasons for lack of sex- and gender-based analysis.*
Population characteristics	*Describe the covariate-relevant population characteristics of the human research participants (e.g. age, genotypic information, past and current diagnosis and treatment categories). If you filled out the behavioural & social sciences study design questions and have nothing to add here, write "See above."*
Recruitment	*Describe how participants were recruited. Outline any potential self-selection bias or other biases that may be present and how these are likely to impact results.*
Ethics oversight	*Identify the organization(s) that approved the study protocol.*

Note that full information on the approval of the study protocol must also be provided in the manuscript.

Field-specific reporting

Please select the one below that is the best fit for your research. If you are not sure, read the appropriate sections before making your selection.

☐ Life sciences ☐ Behavioural & social sciences ☒ Ecological, evolutionary & environmental sciences

For a reference copy of the document with all sections, see nature.com/documents/nr-reporting-summary-flat.pdf

Ecological, evolutionary & environmental sciences study design

All studies must disclose on these points even when the disclosure is negative.

Study description	In this study, we measure the nitrogen isotope composition of organic matter preserved in the tooth enamel (δ15Nenamel) of modern and fossil mammals.
Research sample	We measured δ15Nenamel in modern mammals (n = 54) from several different ecosystems across Africa. In addition, paired δ15Nbone-collagen values were measured for a subset (n = 33) of the same individuals. We also measured fossil mammalian teeth from a Late Pleistocene (38.4–13.4 ka) terrestrial assemblage from Tam Hay Marklot Cave (THM), Laos in southeast (SE) Asia.
Sampling strategy	We targeted herbivores (including browsers, grazers, and mixed feeders), omnivores, and carnivores to evaluate the effect of trophic level on δ15N values. Isotopic datasets were evaluated to determine if the data was normally distributed and if variance was equal between groups using Levene's test. A non-parametric Kruskal-Wallace test followed by a Dunn's post-hoc test with a Bonferroni correction for pairwise comparisons was used when the requirements for ANOVA were not satisfied, otherwise Analysis of variance (ANOVA; one-tailed) was used to identify statistically significant differences in isotopic values between groups for modern African fauna. Where ANOVA indicated statistical significance, pairwise comparisons were made using a Tukey-Kramer HSD post-hoc test to determine which groups differed from one another in their isotope values. Statistically significant relationships between paired isotopic values from the same individuals were determined using Pearson's correlation (two-tailed), Kendall Rank correlation, or Spearman Rank correlation (two-tailed) where appropriate. Statistical analyses were performed using Paleontological Statistics Version 4.09 (PAST4) and JMP[illegible], Version 16 statistical software using an alpha level for significance of 0.05.
Data collection	Data were collected from March 2019 - December 2020.
Timing and spatial scale	Data were collected continuously during this period.
Data exclusions	No data were excluded from analysis.
Reproducibility	Samples were measured in duplicate or triplicate (resulting in a total of 115 individual measurements) and in separate batches.
Randomization	Herbivores, including browsers (consuming mainly C3-plants), grazers (consuming mainly C4-plants), and mixed feeders (consuming both C3- and C4-plants), as well as omnivores and carnivores were selected to ensure that different trophic levels and feeding behaviors were represented.
Blinding	Samples were assigned an analytical number for isotopic analysis.

Did the study involve field work? ☐ Yes ☒ No

Reporting for specific materials, systems and methods

We require information from authors about some types of materials, experimental systems and methods used in many studies. Here, indicate whether each material, system or method listed is relevant to your study. If you are not sure if a list item applies to your research, read the appropriate section before selecting a response.

Materials & experimental systems

n/a	Involved in the study
☒	☐ Antibodies
☒	☐ Eukaryotic cell lines
☐	☒ Palaeontology and archaeology
☐	☒ Animals and other organisms
☒	☐ Clinical data
☒	☐ Dual use research of concern

Methods

n/a	Involved in the study
☒	☐ ChIP-seq
☒	☐ Flow cytometry
☒	☐ MRI-based neuroimaging

Palaeontology and Archaeology

Specimen provenance	From the excacation of Tam Hay Marklot cave site, Huà Pan province, Laos in 2015
Specimen deposition	Ministry of Information, Culture and Tourism, Vientiane, Laos
Dating methods	Uranium-Thorium method on teeth publihed in Bourgon et al., PNAS 2020

☐ Tick this box to confirm that the raw and calibrated dates are available in the paper or in Supplementary Information.

Ethics oversight	*Identify the organization(s) that approved or provided guidance on the study protocol, OR state that no ethical approval or guidance was required and explain why not.*

Note that full information on the approval of the study protocol must also be provided in the manuscript.

Animals and other research organisms

Policy information about studies involving animals; ARRIVE guidelines recommended for reporting animal research, and Sex and Gender in Research

Laboratory animals	The study did not involve laboratory animals.
Wild animals	Isotopic analyses were conducted on the tooth enamel and bone collagen of 20 modern African mammalian taxa housed in the zoological collection at the University of Hamburg, Germany.
Reporting on sex	No sex-based analyses were performed. Sex-based analyses were not relevant to this study.
Field-collected samples	Samples were not collected from the field.
Ethics oversight	No ethical approval or guidance was required for this study.

Note that full information on the approval of the study protocol must also be provided in the manuscript.
